# Acute low back pain as infusion-related reaction to monoclonal antibodies

**DOI:** 10.3389/fonc.2023.1161818

**Published:** 2023-10-24

**Authors:** Pawel Majenka, Carmen Loquai, Tilman Schöning, Alexander Enk, Jessica Hassel

**Affiliations:** ^1^ Department of Dermatology and National Center for Tumor Diseases, University Hospital Heidelberg, Heidelberg, Germany; ^2^ Department of Dermatology, University Medical Center Mainz, Mainz, Germany; ^3^ Department of Pharmacy, University Hospital Heidelberg, Heidelberg, Germany

**Keywords:** skin cancer, immunotherapy, PD-1/PD-L1 inhibitors, infusion-related reactions, low back pain

## Abstract

Monoclonal antibodies, such as PD-1 inhibitors, are increasingly used in various cancers. Acute low back pain as infusion-related reaction (IRR) to monoclonal antibodies is poorly described. We report a bicentric series of 10 cases of acute low back pain due to administration of monoclonal antibodies directed against PD-1/PD-L1 for skin cancer treatment in patients treated at University Hospital Heidelberg and University Medical Center Mainz (Germany). The management of IRR symptoms was immediate interruption of infusion and analgesia leading to quick improvement and complete symptom relief in all patients. Our findings suggest that the risk of developing low back pain as IRR is depending on the concentration of the administered drug. Low back pain as IRR can be managed by early interruption of infusion and by decreasing the infusion rate or concentration in following administrations.

## Introduction

Monoclonal antibodies, such as programmed cell death protein 1 (PD-1) inhibitors, are among the main therapeutic agents for treating melanoma and other cancer entities. Their use can lead to long-term remission, but also risks generating adverse events including infusion-related reactions (IRRs) (1). IRRs usually occur during or shortly after intravenous administration of the drug and range from slight discomfort to life-threatening symptoms. Among the heterogenous symptoms of IRRs to monoclonal antibodies low back pain is described (2), however, there is little published information on this subject and the physiologic correlate of the pain sensation remains unclear. We propose the hypothesis that the acute low back pain is an ischemic pain due to intravascular cell aggregates in the blood vessels.

## Materials and methods

We report a series of 10 cases of acute low back pain due to administration of monoclonal antibodies directed against PD-1/PD-L1 for skin cancer treatment (**Table 1**). Eight patients received nivolumab for stage III (n = 4) and stage IV (n = 4) melanoma. Nivolumab was administered as monotherapy or in combination therapy with ipilimumab or relatlimab. One patient received pembrolizumab for stage IV melanoma, another patient received avelumab for stage IV merkel-cell carcinoma. The present study was prepared in line with journal guidelines for Brief Research Reports and the recommendations of the STROBE (Strengthening the Reporting of Observational studies in Epidemiology) initiative.

**Table 1:** Patient and treatment characteristics.

No	Sex	Age	Diagnosis	Treatment	First IR	Target	Therapy cycle	Total infusion time (min)	Total dosage (mg)	Total volume (ml)	Concen- tration (mg/ml)	Mass flow (mg/min)	Onset of IR (min)	Administered dosage until IR (mg)	Measures which allowed continuation of therapy (without IRR reoccurrence)	Total IgE (IU/ml)	Best tumor response
1	F	77	Stage IV melanoma	Ipilimumab + Nivolumab	Nivolumab 1 mg/kg	PD-1	2	30	77	57.7	1.33	2.56	30	77	none	22	PR
2	M	54	Stage III melanoma	Nivolumab (+/- Ipilimumab) CA209-915	Nivolumab 240 mg/480 mg	PD-1	Study week 5	30	240/480	100	2.4/4.8	8/16	15	120/240	Infusion flow rate reduced by half	20	n.a.
3	F	56	Stage IV melanoma	Ipilimumab + Nivolumab	Nivolumab 1 mg/kg	PD-1	2	30	60	56	1.07	2	23	46	none	<1	PD
4	M	53	Stage III melanoma	Nivolumab (+/- Ipilimumab) CA209-915	Nivolumab 240 mg/480 mg	PD-1	Study week 5	30	240/480	100	2.4/4.8	8/16	20	160/320	Infusion flow rate reduced by half	ND	n.a.
5	M	70	Stage IV melanoma	Pembrolizumab	Pembrolizumab 2mg/kg	PD-1	11	30	290	111.6	2.59	9.6	15	145	none	230	PR
6	M	80	Stage IV melanoma	Ipilimumab + Nivolumab	Nivolumab 1 mg/kg	PD-1	2	60	95	59.5	1.59	1.58	45	71.1	none	ND	n.a.
7	M	51	Stage IV melanoma	Nivolumab (+/- Relatlimab) CA224-047	Nivolumab 480 mg (+/- Relatlimab 160 mg)	PD-1	2	60	480 (+/- Relatlimab 160mg)	100	4.8	8	30	240	none	121	PD
8	M	63	Stage III melanoma	Nivolumab	Nivolumab 3 mg/kg	PD-1	2	60	270	127	2.13	4.5	25	112.5	Infusion flow rate reduced by half	ND	PD
9	F	83	Stage IV merkel-cell carcinoma	Avelumab	Avelumab 10 mg/kg	PD-L1	1	60	650	250	2.6	10.8	60	650	Infusion flow rate reduced by half	ND	CR
10	M	58	Stage III melanoma	Nivolumab	Nivolumab 3 mg/kg	PD-1	Unknown	60	255	100	2.55	4.25	Unknown (during infusion)	Unknown	Infusion flow rate reduced by half	ND	PD

IR, infusion reaction; PCDLBCL, primary cutaneous diffuse large B-cell lymphoma; n. a., not applicable; ND, not done.

## Results

The median time from infusion start to the onset of low back pain was 25 minutes (15-60 minutes). Apart from acute low back pain in all patients (described as pain either in the central, kidney or flank region) concomitant symptoms included abdominal pain (n = 2), pain radiating in the legs (n = 1) or the neck (n = 1). Pain sensation was described as pulsating, colic like, cramping and pressure. Routine laboratory assessment prior to therapy showed in none of the patients irregularities. Serum concentration of total IgE was in the normal range in 3 and slightly elevated in 2 patients (#5, #7). Abdominal ultrasound imaging performed in one patient (#3) showed no abnormalities. None of the patients showed either any remarkable comorbidities predisposing to low back pain or a history of acute low back pain in the past. The management of IRR symptoms was immediate interruption of infusion and analgesia leading to quick improvement and eventually within minutes complete symptom relief in all patients. No steroid treatment or antihistaminics were needed. In 5/10 patients infusions could be resumed with a slower flow rate without reoccurrence of symptoms. Few patients however developed a second (n = 5) or a third (n = 3) onset of equal IRR symptoms in subsequent drug administrations, in 3 cases (#2, #4, #8) even despite a reduction of the flow rate. Half of the patients (n = 5) developed low back pain during the second administration of the antibody. One patient (#6) showed an IRR to the first administration of pembrolizumab after having reacted to nivolumab already before.

The first case of acute low back pain as IRR to monoclonal PD-1/PD-L1 directed antibodies we observed at our department dates august 2017, whereas we did not observe a single case of infusion-related low back pain in the previous years. After consulting with our Department of Pharmacy we noticed that in the case of nivolumab, just one month before the first onset of infusion-related low back pain at our department, the concentration of the active substance has been increased by volume reduction of the carrier solution in the manufacturing process. In all cases the substance was dissolved in sodium chloride 0.9%. All administrations took place through an intravenous line containing a sterile in-line filter with a pore size of 0.2 µm. Concentrations before and after the shift were at all times within the eligible range and thus lege artis. Even after the increase, the concentrations were still far away from the maximum dose of 10 mg/ml, which is allowed according to the prescribing information of the drug. Despite being in the eligible range, the first cases of infusion-related low back pain in our department occurred not until the drug concentration had been increased. This suggests that the risk of developing low back pain as IRR is depending on the concentration of the administered drug. However, in none of the patients we did intentionally change concentration, flow rate or infusion volume just before the cycle during which the IRR occurred for the first time.

## Discussion

The pathophysiology of IRRs is heterogenous. Immediate and nonimmediate hypersensitivity reactions (HSRs) to monoclonal antibodies have been described (3), among them IRRs, which in turn may be defined as symptoms experienced by patients during the infusion or within 24 hours from the first administration of the monoclonal antibody, although they appear most frequently from 10 minutes to 4 hours after beginning the administration. The literature also distinguishes between different types of HSRs, including IgE-mediated and non-IgE reactions, anaphylaxis, allergic reactions and cytokine-release syndrome. Clinical manifestations of these reactions vary and the symptoms of all types of immunologically mediated infusion reactions overlap, making it challenging for the clinician to identify the cause. As there are IgE- and non-IgE-HSRs, we found it interesting to include IgE-levels in our patients. IgE was slightly elevated only in two of our patients (**Table 1**) which does not allow any conclusions. Further investigation is needed for this matter. Besides flushing, rash, dyspnea, chest pain and subjective distress, the symptom of back pain is specifically listed among non-IgE type complement activation-related pseudoallergy (CARPA) IRRs, which are caused by several liposomal and micellar formulations, nanoparticles, radiocontrast agents, and therapeutic antibodies and typically occur within minutes after the start of the infusion. CARPA has been reported as a HSR, “the symptoms of which fit into the Coombs and Gell’s Type I category, which is not initiated or mediated by pre-existing IgE antibodies but arises as a consequence of activation of the complement system” (2). As with all biologic agents, there is an inherent risk of developing antidrug antibodies (ADAs) during repeated infusions. The consequences of immunogenicity range from the absence of a clinical effect to severe, life‐threatening events including infusion reactions. However, there is no evidence that the incidence of HSRs or IRRs is associated with the presence of antidrug antibodies (ADAs) (4).

Our aim was to report a series of patients with acute low back pain after administration of monoclonal antibodies specifically directed against PD-1/PD-L1. All cases (n=10) that we observed between the first appearance in 2017 and 2018 have been included in the study. Interestingly, our case series shows that symptoms appeared in patients having received different types of monoclonal antibodies directed against PD-1/PD-L1 and symptoms did not appear only after administration of a specific antibody like nivolumab. Nivolumab, pembrolizumab and avelumab are all antibodies targeting PD-1/PD-L1 pathway, which makes them somewhat comparable not only in mode of action but also in spectrum of side effects. Thus, although these drugs are different substances, they are from the same substance group. In contrast, we have not seen similar reactions after administration of e.g. the CTLA-4 antibody ipilimumab alone. However, the type of antibody is different also: Nivolumab and pembrolizumab are IgG4 antibodies, whereas ipilimumab and avelumab are IgG1 type. However, nivolumab was administered much more frequently than pembrolizumab or avelumab in the centers participating in the study at that time, which is probably the reason for having seen more symptoms in patients who received nivolumab than in patients who received pembrolizumab or avelumab.

Similar cases of acute devastating low back pain have also been described during infusions of other agents such as amiodarone (5, 6). Petrou et al. observed the occurrence of epigastric and low back pain without specific clinical signs of histamine secretion (lips swelling, upper respiratory tract edema and skin manifestations) and thus concluded that in their patients no classical anaphylaxis was the case. As a physiologic correlate for the epigastric and low back pain the authors propose a hypotensive response that results in transient secondary mesenteric ischemia (5). Korantzopoulos et al. discuss an allergic or idiosyncratic reaction as in their cases the lumbar symptoms were accompanied with a pruritic rash in one case and with presyncopal symptoms in another case (6).

Some publications discuss drug safety in the context of IRRs. Choi et al. report a patient with hepatocellular carcinoma successfully treated with pembrolizumab after experiencing an IRR with flushing, dyspnea and back pain to nivolumab (7). In our case (#6) however, the same IRR reoccurred after switching from nivolumab to pembrolizumab. Rombouts et al. conclude from their review on infusion reactions and infusion rates of monoclonal antibodies that administration of certain antibodies (bevacizumab, ipilimumab, low dose nivolumab, panitumumab and rituximab) in an increased infusion rate as compared to the one stated by the manufacturer is safe (8). Nevertheless, our observations suggest that clinicians should alter infusion rates with care. Not only did we observe symptom relief after slowing infusion rates in most patients, but also we have seen higher rates of IRRs after increasing the antibody concentration in the infusion while still being within the limits stated by the manufacturer.

The cause of the side effect acute low back pain remains unclear. We assume, that the observed clinical manifestations do not fall within the category of typical immune reactions. Since dosage seems to be a crucial factor, we believe that decreasing the infusion rate or concentration presumably leads to less accumulation of intravascular cell aggregates in blood vessels and thereby prevents the development of ischemic pain sensations.

In conclusion, acute low back pain as IRR to monoclonal antibodies is rare and the pathophysiology is not yet thoroughly understood. The risk of severe infusion induced pain possibly leading to therapy drop-out requires clinicians to pay attention to such symptoms in the course of intravenous drug administration. The onset of low back pain during or shortly after infusion, the acute progression and the severity of symptoms support the diagnosis of an IRR. Our series suggests that low back pain as IRR can be managed by early interruption of infusion and by decreasing the infusion rate or concentration in following administrations, while discontinuation of therapy is not strictly required in most cases.

## Data availability statement

The original contributions presented in the study are included in the article/supplementary material. Further inquiries can be directed to the corresponding author.

## Ethics statement

The studies involving humans were approved by the institutional review board of the Medical Faculty of the Heidelberg University Hospital (approval number S-454/2015). The studies were conducted in accordance with the local legislation and institutional requirements. Written informed consent for participation was not required from the participants or the participants’ legal guardians/next of kin in accordance with the national legislation and institutional requirements. Written informed consent was not obtained from the individual(s) for the publication of any potentially identifiable images or data included in this article because the ethical committee of the institutional review board of the Medical Faculty of the Heidelberg University Hospital had agreed to the retrospective analysis of routinely collected clinical data without prior informed consent of patients.

## Author contributions

PM and JH contributed to conception and design of the study. PM, JH and CL organized the database. PM, JH and TS participated in the final data analysis and interpretation. PM wrote the first draft of the manuscript with input from other authors. All authors contributed to manuscript revision, read, and approved the submitted version.
